# Exploring hormonal influences on nicotine craving and use across the perinatal period: A prospective longitudinal study

**DOI:** 10.1016/j.dadr.2026.100469

**Published:** 2026-08-13

**Authors:** Alicia M. Allen, James Baurley, Linnea B. Linde-Krieger, Arushi Chalke, Whitney Norris, Xinger Xia, Ashley H. Clawson

**Affiliations:** a National Center for Opioid and Addiction Research, Arkansas Children’s Research Institute, 13 Children’s Way, Slot 842, Little Rock, AR 72202, United States; b Department of Pediatrics, College of Medicine, University of Arkansas for Medical Sciences, 1300 South Medical Drive, Little Rock, AR 72202, United States; c BioRealm LLC, 19330 Rim of the World Drive, Monument, CO 80132, United States; d School of Social Work, Arizona State University, 411 N Central Ave, #800, Phoenix, AZ 85004, United States; e Epidemiology and Biostatistics, Mel and Enid Zuckerman College of Public Health, University of Arizona, Tucson, AZ, USA; f College of Nursing, University of Arkansas for Medical Sciences, 4301 W Markham St, Little Rock, AR 72205, United States; g Department of Health Behavior and Health Education, Fay W. Boozman College of Public Health, University of Arkansas for Medical Sciences, 4301 W Markham St, Little Rock, AR 72205, United States

**Keywords:** Perinatal, Nicotine, Hormones, Bayesian

## Abstract

**Introduction:**

Perinatal nicotine use is common despite well-documented adverse consequences. We examined associations between reproductive-related hormones with nicotine craving and use during the perinatal period to identify potential novel intervention points.

**Methods:**

All participants reported use of nicotine during the perinatal period. Participants were enrolled at gestational week ≥ 36 and followed to postpartum week 12 via daily surveys (i.e., nicotine craving via 100-point scale, dichotomous use) and weekly hormone measurement in saliva (cortisol, oxytocin) or dried blood spots (progesterone, estradiol, testosterone, dehydroepiandrosterone sulfate). Bayesian mixed-effects models accounted for within-person correlation while estimating hormone effects.

**Results:**

Participants (n = 46) were 28.9 ± 4.9 years old. During follow-up, exclusive combustible cigarettes (n = 20), electronic nicotine delivery systems (ENDS; n = 13), or dual (n = 2) use was observed, with variability in use and craving across participants and over time. During pregnancy, higher oxytocin was linked to greater craving (β=16.31, 95% CI: 3.71, 28.83). Greater peripartum declines in oxytocin were associated with more craving (β=8.71, 95% CI: 0.75, 16.93) and use (β=1.13, 95% CI: 0.05, 2.43). During postpartum, lower estradiol was linked to more craving (β=-1.17, 95% CI: −2.15, −0.18) and use (β=-0.40, 95% CI: −0.76, −0.03). In models simultaneously evaluating all postpartum hormones, the lone meaningful association was between estradiol and craving (β=-1.66, 95% CI: −2.84, −0.48).

**Conclusions:**

The results of this study suggest that oxytocin and estradiol may contribute to the risk of perinatal nicotine use. Additional research is needed to replicate our observations in more diverse study samples and explore implications for clinical intervention.

## 1 Introduction

From 2016–2021 in the United States (US), the prevalence of combustible cigarette (CC) use during pregnancy dropped by a staggering 33% (Martin et al., 2023), which was the first statistically significant decline in prevalence in decades (Rockhill et al., 2016). However, concurrently, the prevalence of use of electronic nicotine delivery systems (ENDS) during pregnancy increased two- to four-fold (Beckodro et al., 2026, Head et al., 2022, Wen et al., 2023). Consequently, despite the shifts in product popularity, the use of nicotine during the perinatal period persists as a highly prevalent phenomenon. Current estimates indicate that 12% of pregnant people in the US report nicotine use, with 5% reporting CC use only, 5% using ENDS only, plus 2% reporting dual use of CC and ENDS (Masud et al., 2024). Regardless of source, nicotine exposure during pregnancy has adverse effects on fetal development (Deprato et al., 2025a, SECTION ON NICOTINE AND TOBACCO PREVENTION AND TREATMENT, C.O.S.U.A.P., SECTION ON NICOTINE AND TOBACCO PREVENTION AND TREATMENT, C.O.S.U.A.P., 2023) and pregnancy outcomes (Deprato et al., 2025b, SECTION ON NICOTINE AND TOBACCO PREVENTION AND TREATMENT, C.O.S.U.A.P., SECTION ON NICOTINE AND TOBACCO PREVENTION AND TREATMENT, C.O.S.U.A.P., 2023). Further, among those who abstain from nicotine use during pregnancy, return to nicotine use after childbirth is common with more than a third returning to nicotine use within three months after delivery (Bowker et al., 2021, De Genna et al., 2023, Lauren Kipling et al., 2024). Passive exposure to CC adversely impacts children via increased risks of acute (e.g., bronchiolitis, ear infections) and chronic (e.g., asthma, cancer) health issues (Jenssen et al., 2023). While the evidence for the link between passive exposure to aerosol produced from ENDS and adverse health consequences in children is still developing, current reports indicate that this aerosol contains many of the same harmful toxins as secondhand smoke and is linked to both nicotine absorption and deleterious health outcomes among children (e.g., asthma, ear infections) (Dai et al., 2025, SECTION ON NICOTINE AND TOBACCO PREVENTION AND TREATMENT, C.O.S.U.A.P., SECTION ON NICOTINE AND TOBACCO PREVENTION AND TREATMENT, C.O.S.U.A.P., 2023, Rodriguez et al., 2026). Reducing the use of CC and/or ENDS during the perinatal period will reduce adverse health consequences in both mothers and children. Understanding the drivers of perinatal nicotine use can directly inform the development of novel cessation and/or return-to-use prevention efforts.

Reproductive-related hormones may play an important role in perinatal nicotine craving and use. Progesterone, a hormone that plays an essential role in both establishing and maintaining pregnancy, has been implicated in protecting against nicotine use (Lynch and Sofuoglu, 2010, Peltier and Sofuoglu, 2018). In double-blind randomized control trials, exogenous administration of progesterone has also been linked to improved CC cessation outcomes in non-pregnant women of reproductive age (Tosun et al., 2019). Additionally, exogenous progesterone shows promise in both blunting nicotine craving, as well as the prevention of postpartum return to smoking in two separate double-blind randomized pilot studies (Allen et al., 2018, Allen et al., 2016, Forray et al., 2017), with an efficacy trial now underway (Abdelwahab et al., 2024). While less is known about the link between progesterone and ENDS use, estradiol has been linked to an increase in ENDS use among naturally-cycling females (Vergara-Lopez et al., 2025). This observation aligns with the hypothesis that estradiol enhances the favorable effects of nicotine (Wetherill et al., 2016), suggesting that the neurobiological reward response to nicotine delivered via ENDS may be similar to that observed in CC delivery. These hormonal contributions to nicotine use may be especially salient during the transition from pregnancy to postpartum given the corresponding dramatic hormonal decline (Yen, Jaffe’s, 2023). Moreover, emerging evidence indicates that the change in hormone levels may be a stronger predictor of nicotine use than absolute hormone levels (Saladin et al., 2015), though this remains an underexplored area.

Beyond progesterone and estradiol, few studies have examined associations among nicotine use and the numerous other reproductive-related hormones that have a known metabolic link to progesterone and/or estrogen, as well as a known influence on neurobiological reward response. For example, oxytocin stimulates breastfeeding and is higher during lactation (Walter et al., 2021a). Interestingly, breastfeeding is a well-documented protective factor against postpartum CC use (Linde-Krieger et al., 2026, Orton et al., 2018), yet the possible hormonal underpinnings of this observation remain unexplored. Oxytocin also promotes maternal-infant bonding (Walter et al., 2021b) and bonding impairment is linked to postpartum CC (Faisal-Cury and Matijasevich, 2022, Phillips et al., 2012). Oxytocin has been recently implicated as a possible intervention point given the observed favorable effects on craving, withdrawal, and stress (Mellentin et al., 2023, Ren et al., 2024, Thanekar et al., 2026). Even less is known about testosterone and dehydroepiandrosterone sulfate (DHEAS), though their influence on drug taking behaviors also has biological plausibility (Yadid et al., 2010). Finally, stress-related hormones, namely cortisol, have been implicated in CC use, with higher cortisol linked to both the subjective motives for smoking (e.g., affect, withdrawal) and return-to-use risk; yet relationships may vary by sex and menstrual phase, suggesting that reproductive-related hormones may alter the effect of stress hormones on nicotine use (al’Absi et al., 2015, Carlson et al., 2017, Huttlin et al., 2015). Importantly, these hormones share metabolic pathways, yet nearly all research to date has examined the influence of a single hormone in isolation from other hormones. The lone exception is exploration of the progesterone to estradiol (P:E2) ratio, which has been documented to have an inverse association with craving in some (Harrison et al., 2020, Pang et al., 2018), but not all (Harrison et al., 2025, Schick et al., 2024) research. In sum, while there is some evidence that reproductive-related hormones influence nicotine use, particularly CC use, numerous hormones have yet to be examined either individually or concurrently and these associations have yet to be examined within ENDS use or during the perinatal period.

This secondary data analysis seeks to explore the associations between perinatal nicotine craving and use with hormones using both subject-level and repeated-measures analyses. Specifically, we aimed to examine the link between static hormonal levels and changes across the transition from pregnancy to postpartum with craving and use of nicotine. By examining hormones during late pregnancy (≥ 36 weeks gestation), early postpartum (weeks 1–12), and the change from late pregnancy to early postpartum, this investigation will fill current gaps in knowledge with regard to hormonal influences on perinatal nicotine use (i.e., expanding from CC use to CC and/or ENDS use), exploration of new hormones (i.e., oxytocin, testosterone, DHEAS), and a holistic examination of these hormones together. Ultimately, understanding how hormones influence nicotine craving and use during the perinatal period can inform effective cessation and return-to-use prevention interventions tailored to this unique period.

## 2 Methods

### 2.1 Study overview

This secondary data analysis utilized data from a prospective cohort study that enrolled participants with and without opioid use disorder with a goal of identifying novel intervention targets for opioid use disorder recovery support specific to the early postpartum period (Allen et al., 2024). Participants completed the baseline visit during pregnancy at gestational week 36 or beyond. The day after baseline, participants who reported a recent history of nicotine use commenced daily measurement of nicotine craving and nicotine use. In brief, these data were collected via daily surveys launched each evening at 8 pm. After childbirth, participants commenced weekly visits continuing through 12 weeks postpartum. During the baseline and follow-up visits, participants met with study staff to complete the retrospective report of nicotine use via the TimeLine FollowBack (TLFB) interview (Robinson et al., 2014) and to collect saliva and dried blood spot samples for the measurement of hormones. All procedures were approved by the University of Arizona’s Institutional Review Board. Additional details can be found elsewhere (Allen et al., 2024).

### 2.2 Study Sample

Primary eligibility criteria of the parent study included: (1) aged 18–40 years, (2) otherwise uncomplicated single-gestation pregnancy, and (3) self-reported expectation to reside with the infant after delivery. Additionally, the parent study enrolled those with opioid use disorder operationalized as opioid use during pregnancy and enrolled in an opioid use disorder treatment program with plans to continue post birth. Control participants were excluded for use of opioid during pregnancy or a history of opioid use disorder. Additional detail about the parent study, eligibility criteria, and the study sample may be found elsewhere (Allen et al., 2024). For the present project, the sample was restricted to those participants who reported nicotine use via CC and/or ENDS at any point during the perinatal period (defined here as at least one puff within three months prior to pregnancy through 12 weeks postpartum). Nicotine use was captured at study enrollment via self-report to four dichotomous self-administered survey questions to capture lifetime use, use within the three months prior to pregnancy, use during pregnancy, and current use.

### 2.3 Study measures and timing

Six hormones were assayed once during pregnancy (≥ 36 weeks gestation) and weekly during postpartum weeks 1–12: progesterone, estradiol, testosterone, DHEAS, cortisol, and oxytocin. The specific data collection time points were selected to align with the known variation in these hormones in relation to pregnancy, birth, and lactation (Yen, Jaffe’s, 2023). All hormones were collected at standardized times during the day to account for the known diurnal variations. Cortisol was measured twice per day to capture the expected peak (30 min after waking) and nadir (8 pm) levels. The remaining hormones were collected at the study visits between the hours of 7 am and 11 am. Oxytocin was measured via salivary samples whereas progesterone, estradiol, testosterone, and DHEAS were measured via dried blood spots. Cortisol and oxytocin were analyzed at the Laboratory for the Evolutionary Endocrinology of Primates (LEEP) at the University of Arizona. The remaining hormones were analyzed by ZRT Laboratory (Beaverton, OR). Additional details have been reported previously (Allen et al., 2024).

Nicotine craving and binary use (yes/no) were measured daily beginning the day after the baseline visit in late pregnancy through postpartum week 12. Regardless of current nicotine use, nicotine craving was assessed in all participants who reported recent nicotine use at enrollment (i.e., endorsed use within the three months prior to pregnancy, during pregnancy, or currently). This was done using an investigator-created item with a single 100-point Visual Analog Scale (VAS; 0 indicative of ‘not at all,’ 100 indicative of ‘severe’) in response to “Did you crave any of the following today… cigarettes, e-cigarettes, or other forms of tobacco or nicotine?” Nicotine use was prospectively captured dichotomously (use versus no use) via the daily survey. Missing daily survey data were supplemented with the retrospectively reported TLFB data. Using a seven-day point prevalence definition at postpartum week four, a puff or more was defined as use (Piper et al., 2019).

We used three time periods to examine the associations between hormones and nicotine-related variables. First, the prenatal period included values that were collected during late pregnancy (≥ 36 weeks gestation) until childbirth. Second, the postpartum period included data collected from childbirth to 12 weeks postpartum. Third, the change from late pregnancy to early postpartum included data from the late pregnancy through postpartum week four. Postpartum week four was selected as the end of the transitionary stage in an effort to capture the greatest hormone change expected given it is prior to resumption of the menstrual cycle in those who were not breastfeeding (Yen, Jaffe’s, 2023).

Additional variables used to describe the study sample were collected at enrollment (e.g., age, race/ethnicity, parity) using standardized questionnaires.

### 2.4 Statistical analyses

All analyses were conducted using R statistical software (version 4.5)(Team, 2021) using the bpaup R package (Baurley et al., 2025). Hormones were log-transformed prior to analysis to address the right-skew distributions of the hormone values; values below the assay detection limit were replaced with half the detection limit prior to transformation. Two derived predictors were also computed as log-ratios: the P:E2 ratio (log[progesterone] - log[estradiol]) and diurnal cortisol (log[morning cortisol] - log[evening cortisol]), yielding a total of eight hormone variables. Given the candidate set of hormones were related via shared biosynthetic pathways and the longitudinal design with repeated assessments, we employed Bayesian mixed-effects models to account for within-person correlation while estimating time trends and hormone effects. For craving, a continuous outcome, we used an identity link. For use, a binary outcome, we used a logit link. Missing data were handled through complete case analysis within each model. For the mixed-effects craving models, participants were included if they had ≥ 2 observations across ≥ 2 timepoints to support estimation of individual trajectories. For the mixed-effects use models, a stricter threshold of ≥ 5 observations across ≥ 2 timepoints was applied, and participants were further restricted to those with variation in the outcome, as those who report use on every day or on no days contribute no information to the estimation of the hormone effects. For the subject-level regression models, all participants with non-missing data on both the predictor and outcome were included. Each hormone was examined as a separate predictor.

To examine hormone associations with nicotine craving, we fit three model specifications for each hormone. First, we examined subject-level associations between baseline (late pregnancy) hormone values and mean prenatal craving using Bayesian linear regression: mean_craving_i = β₀ + β₁(log_hormone_i) + ε_i. Second, we fit Bayesian linear mixed-effects models to daily postpartum craving (weeks 1–12) with time-varying weekly hormone values: craving_it = β₀ + β₁(study_week_it) + β₂(log_hormone_it) + b₀ᵢ + b₁ᵢ(study_week_it) + ε_it. Third, we examined the association between the change in hormones from late pregnancy to four weeks postpartum (change = log[late pregnancy] – log[postpartum week4], such that positive values indicated a hormonal decline from pregnancy to postpartum) and mean postpartum craving using Bayesian linear regression: mean_craving_i = β₀ + β₁(change_hormone_i) + ε_i.

To examine hormone associations with nicotine use, we fit three model specifications for each hormone. First, we examined the subject-level association between baseline (late pregnancy) hormone values and any prenatal nicotine use observed during daily assessments using Bayesian logistic regression: logit(P(any_use_i = 1)) = β₀ + β₁(log_hormone_i). Second, we fit a Bayesian generalized linear mixed-effects model (logistic) to daily postpartum use (weeks 1–12) with time-varying weekly hormone values: logit(P(use_it = 1)) = β₀ + β₁(study_week_it) + β₂(log_hormone_it) + b₀ᵢ + b₁ᵢ(study_week_it). Third, we examined the association between change in hormones (delta, as above) and seven-day point prevalence of nicotine use at postpartum week four using Bayesian logistic regression: logit(P(use_i = 1)) = β₀ + β₁(delta_hormone_i).

To assess which hormone associations persisted after accounting for collinearity among hormones that share biosynthetic pathways, we fit joint models that included multiple hormone variables simultaneously as predictors in the postpartum craving and used the mixed-effects models described above.

All mixed-effects models included participant-specific random intercepts and slopes to account for individual heterogeneity and time trends. Diffuse priors were specified for all models. For the regression models, fixed effects coefficients were assigned normal priors with variance T² = 100, and error precision (1/σ²) was assigned a Gamma(1,1) prior. For the mixed-effects models, fixed effects coefficients were assigned normal priors with variance T² = 10 (scaled by residual variance), and error precision (1/σ²) and random effects precision were each assigned Gamma(1,1) priors. Continuous-outcome models (craving) were estimated using 25,000 MCMC iterations with 5000 burn-in samples. Use models were estimated using 50,000 MCMC iterations with 5000 burn-in samples. Convergence was assessed via visual inspection of trace plots for all model parameters. Parameter estimates are reported as posterior means with 95% credible intervals (CIs). For logistic models, odds ratios are additionally reported. Those 95% CIs that exclude zero are considered a meaningful association, a common Bayesian decision rule indicating posterior probability strongly favors a non-null effect (Goligher et al., 2024).

## 3 Results

### 3.1 Study participants

Of the 61 participants enrolled in the parent study, 14 participants were excluded from the present analysis due to no reported nicotine use throughout the perinatal period (from three months prior to pregnancy to 12 weeks postpartum). One additional participant was excluded given she reported a single ENDS puff at five weeks postpartum with no other reported nicotine use and, consequently, no data was collected regarding nicotine craving and use. Therefore, a total of 46 participants met eligibility criteria and were included (Table 1). Participants were, on average, 28.8 ± 4.9 years old. Most had opioid use disorder (78%), were multiparous (78**%**) and reported using one nicotine product (72%; 44% exclusive CC use, 28% exclusive ENDS use).

**Table 1:** Study Sample Description (n = 46).

	**Total Sample**	
**Demographics**		
Age	28.8 ± 4.9	
Race/Ethnicity	White, non-Hispanic: 22 (48%) Hispanic: 20 (44%) Other: 4 (9%)	
Education	Some high school or less: 7 (15%) High school diploma or equivalent: 17 (37%) Some college or more: 22 (48%)	
Parent Study Group	Opioid Use Disorder: 36 (78%) Control: 10 (22%)	
	
**Pregnancy-Related**		
Parity (multiparous)	36 (78%)	
Mode of Delivery	Vaginal: 25 (54%) Cesarean: 15 (33%) Missing: 6 (13%)	
Breastfeeding at Week 4 Postpartum	Exclusive: 4 (9%) Combination: 19 (41%) None: 14 (30%) Missing: 9 (20%)	
	
**Nicotine-Related**		
Any Use Three Months Before Pregnancy (yes)	44 (96%)	
Any Use During Pregnancy (yes)	32 (70%)	
Any Use at Study Enrollment (yes)	31 (67%)	
Type of Nicotine Use	Exclusive CC Use: 20 (44%) Exclusive ENDS Use: 13 (28%) Dual Use: 2 (4%) Unknown: 4 (9%) No Observed Use: 7 (15%)	
Amount of Use during Pregnancy ⁎	CC: 5.4 ± 3.5 cigarettes/day ENDS: 27.6 ± 59.6 puffs/day	
Amount of Use during Postpartum Follow-Up	CC: 6.0 ± 3.2 cigarettes/day ENDS: 28.7 ± 41.3 puffs/day	
	
**Hormones**	**Third Trimester** **(n = 37)**	**Postpartum Week 4** **(n = 29)**
Progesterone (ng/mL)	118.0 ± 25.7	0.26 ± 0.3
Estradiol (pg/mL)	20163.9 ± 8925.3	37.17 ± 43.3
Progesterone:Estradiol Ratio (ng/pg)	0.01 ± 0.0	0.02 ± 0.0
Testosterone (ng/dL)	94.8 ± 62.5	20.3 ± 11.1
Dehydroepiandrosterone Sulfate (µg/dL)	70.7 ± 66.3	113.2 ± 104.7
Oxytocin (pg/mL)	75.4 ± 143.8^a^	65.6 ± 58.0^b^
Cortisol @ 30 min after waking (pg/mL)	2922.4 ± 1362.5^c^	1804.9 ± 1717.2^a^
Diurnal Cortisol (pg/mL)	2.7 ± 1.9^c^	8.0 ± 9.7^d^

CC =  Combustible Cigarettes, ENDS =  Electronic Nicotine Delivery System,

^⁎^ From the day after enrollment to childbirth.

^⁎⁎^ Calculated by subtracting value of nadir cortisol (8 pm) value from cortisol value at 30 min after waking.

a n = 31,

b n = 32,

c n = 29,

d n = 30

### 3.2 Associations between hormones & nicotine craving

**Fig. 1:**
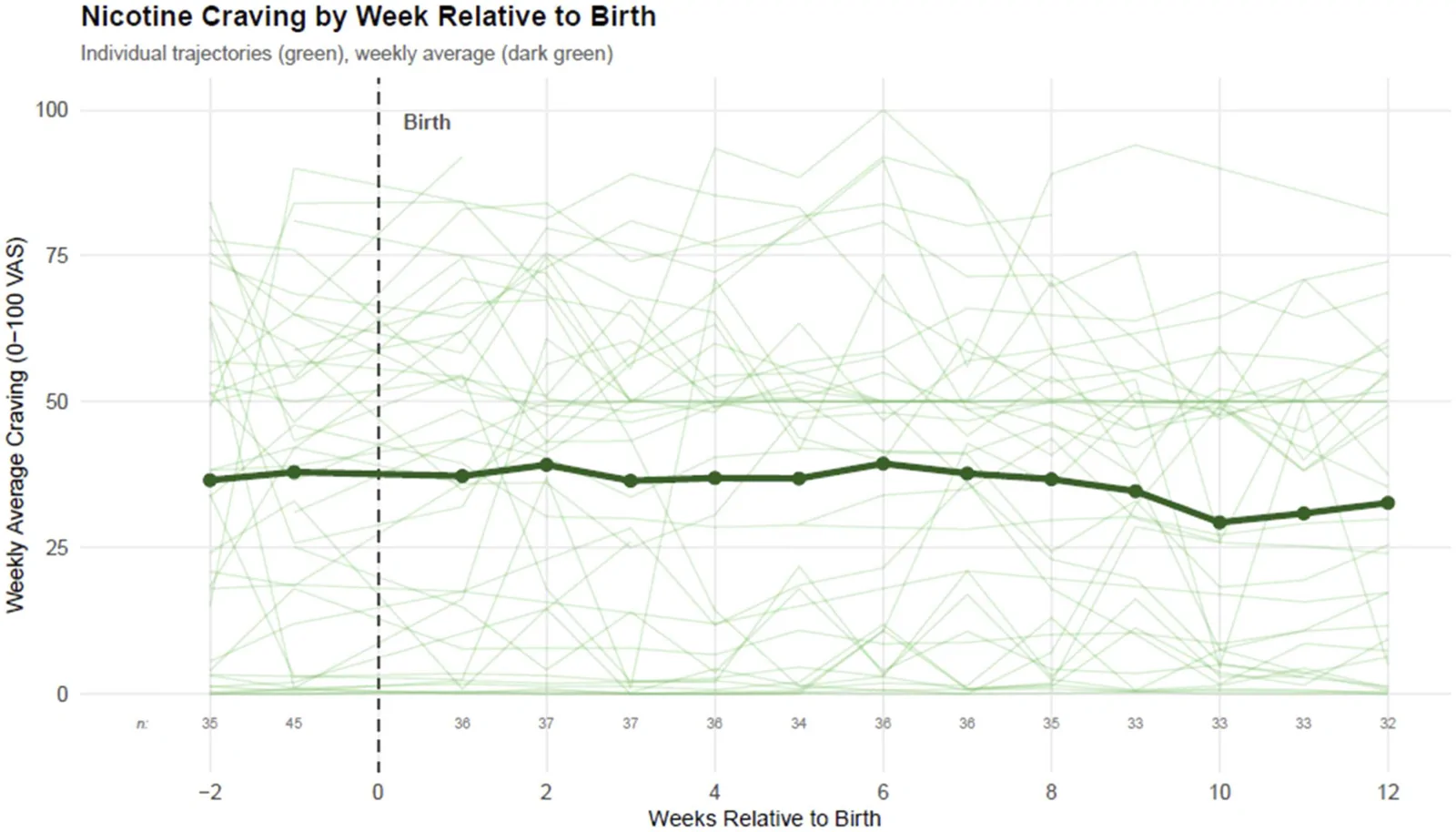
Alt Text: Line graph displaying nicotine craving on a 100-point visual analog scale from 2 weeks before childbirth to 12 weeks post childbirth. Average weekly change: β= −0.24, 95% CI: −0.61, 0.12.

From late pregnancy through postpartum week 12, average group-level craving was stable (β=−0.24, 95% CI: −0.61, 0.12), though individuals demonstrated marked intraindividual variability in daily craving levels (Fig. 1). Prenatally, oxytocin was the only hormone to exert a meaningful influence on craving. Participants with higher oxytocin values at the end of pregnancy reported greater prenatal craving levels (β=16.31, 95% CI: 3.71, 28.83; Table 2). Further, changes in oxytocin levels from pregnancy to postpartum were associated with craving levels, with participants who showed a greater decline in oxytocin from pregnancy to postpartum week four reporting greater postpartum craving (β=8.71, 95% CI: 0.75, 16.93). During postpartum, two hormones were meaningfully associated with craving levels. Specifically, lower estradiol and a higher P:E2 ratio were each associated with higher postpartum craving (β=−1.17, 95% CI: −2.15, −0.18; β=0.87, 95% CI: 0.11, 1.62; respectively). Finally, in a model that included all hormones simultaneously, an inverse association was noted between estradiol and craving (β=−1.66, 95% CI: −2.84, −0.48).

**Table 2:** Association Between Hormones & Nicotine Craving (β, 95% Credible Interval).

	**Prenatal Only**^a^ **(n = 36)**	**Change from Pregnancy to Postpartum Week 4**^a^, ^c^ **(n = 29)**	**Postpartum Only**	
			**Single Hormone Models**^b^ **(n = 37)**	**Combined Hormone Model**^b^ **(n = 32)**
Progesterone	−21.15 (−59.69, 17.66)	−4.36 (−17.53, 8.79)	0.28 (−0.68, 1.26)	0.19 (−0.81, 1.20)
Estradiol	−9.61 (−27.85, 8.58)	2.25 (−4.65, 9.16)	**−1.17 (−2.15, −0.18) ⁎**	**−1.66 (−2.84, −0.48) ⁎**
Progesterone:Estradiol Ratio	6.10 (−13.73, 25.79)	−4.26 (−11.69, 3.30)	**0.87 (0.11, 1.62) ⁎**	-
Testosterone	2.34 (−12.02, 16.49)	1.36 (−9.98, 12.75)	−0.02 (−2.15, 2.11)	1.00 (−1.60, 3.60)
Dehydroepiandrosterone Sulfate	−2.76 (−12.87, 7.35)	3.41 (−14.05, 21.09)	1.56 (−1.88, 5.01)	3.88 (−0.19, 7.96)
Oxytocin	**16.31 (3.71, 28.83) ⁎**^e^	**8.71 (0.75, 16.93) ⁎**	−0.01 (−1.11, 1.07)^f^	−0.05 (−1.22, 1.11)
Cortisol @ 30 min after waking	−16.53 (−34.40, 1.39)^g^	6.13 (−4.84, 17.39)^i^	0.27 (−0.81, 1.35)^h^	−0.25 (−1.56, 1.05)
Diurnal Cortisol^d^	−6.73 (−22.93, 9.41)^g^	4.47 (−2.05, 11.06) j	−0.05 (−0.83, 0.75)^f^	-
Cortisol @ 8 pm	-	-	-	0.45 (−0.54, 1.43)

^⁎^Zero outside of the 95% credible interval.

a Between-subject analyses.

b Repeated measures mixed-effect analyses.

c The hormone value was calculated by subtracting the log of the postpartum week four value from the log of the gestation week ≥ 36 value.

d Calculated by subtracting value of the log of the nadir cortisol (8 pm) value from the log of the cortisol value at 30 min after waking.

e n = 30

f n = 34

g n = 28

h n = 35

i n = 24

j n = 27

### 3.3 Associations between hormones & nicotine use

**Fig. 2:**
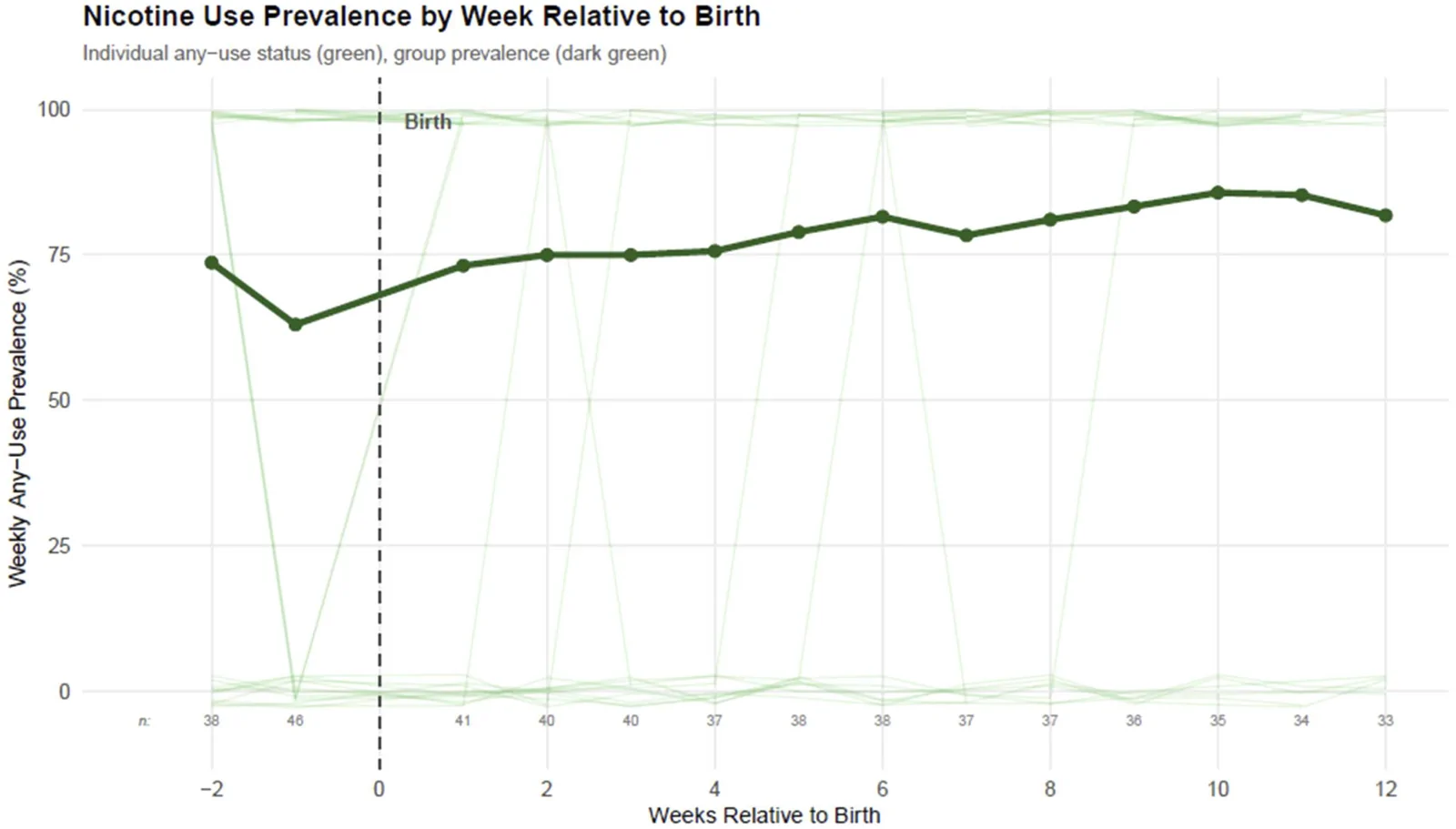
Alt Text: Line graph displaying prevalence of nicotine use by week from 2 weeks before childbirth to 12 weeks post childbirth. Average weekly change: β= 0.63, 95% CI: 0.47, 0.82.

Overall, the prevalence of nicotine use increased from late pregnancy to postpartum week 12 (β=0.63, 95% CI: 0.47, 0.82; Fig. 2). Prenatally, cortisol was the only hormone that was associated with use, such that participants with lower morning cortisol were more likely to report nicotine use (β=−1.58, 95% CI: −3.26, −0.04; Table 3). Regarding hormone changes from pregnancy to the postpartum, participants who had a greater decline in oxytocin from late pregnancy to four weeks postpartum were more likely to report nicotine use at postpartum week four (β=1.13, 95% CI: 0.05, 2.43). During postpartum, lower estradiol was associated with greater nicotine use (β=−0.40, 95% CI: −0.76, −0.03). Finally, in a model that included all hormones simultaneously (n = 13), no hormones had a meaningful association with use.

**Table 3:** Association Between Hormones & Nicotine Use (β, 95% Credible Interval).

	**Prenatal Only**^a^ **(n = 37)**	**Change from Pregnancy to Postpartum Week 4**^a^, ^c^ **(n = 29)**	**Postpartum Only**	
			**Single Hormone Models**^b^ **(n = 15)**	**Combined Hormone Model**^b^ **(n = 13)**
Progesterone	−0.77 (−3.51, 1.99)	−0.71 (−2.32, 0.69)	0.01 (−0.29, 0.31)	−0.03 (−0.41, 0.35)
Estradiol	−0.40 (−1.73, 0.91)	0.18 (−0.55, 0.94)	**−0.40 (−0.76, −0.03) ⁎**	−0.39 (−0.88, 0.11)
Progesterone:Estradiol Ratio	0.37 (−1.26, 2.07)	−0.51 (−1.50, 0.36)	0.21 (−0.04, 0.47)	-
Testosterone	−0.40 (−1.64, 0.79)	−0.34 (−1.64, 0.89)	−0.45 (−1.13, 0.22)	−0.47 (−1.61, 0.64)
Dehydroepiandrosterone Sulfate	−0.15 (−1.09, 0.76)	−1.24 (−3.60, 0.82)	−0.01 (−0.81, 0.85)	−0.39 (−1.71, 1.02)
Oxytocin	1.23 (−0.16, 2.83)^e^	**1.13 (0.05, 2.43) ⁎**^g^	0.46 (−0.75, 1.65)^f^	0.04 (−0.48, 0.58)
Cortisol @ 30 min after waking	**−1.58 (−3.26, −0.04) ⁎**^h^	0.64 (−0.57, 1.95) j	−0.14 (−0.54, 0.26)^i^	0.29 (−0.30, 0.89)
Diurnal Cortisol^d^	−1.23 (−2.99, 0.31)^h^	0.46 (−0.28, 1.27) k	−0.13 (−0.46, 0.20)	-
Cortisol @ 8 pm	-	-	-	0.24 (−0.19, 0.67)

⁎ Zero outside of the 95% credible interval.

a Between-subject analyses.

b Repeated measures mixed-effect analyses restricted to those who had variability in yes versus no use.

c The hormone value was calculated by subtracting the log of the postpartum week four value from the log of the gestation week ≥ 36 value.

d Calculated by subtracting value of the log of the nadir cortisol (8 pm) value from the log of the cortisol value at 30 min after waking.

e n = 31

f n = 16

g n = 27

h n = 29

i n = 31

j n = 24

k n = 27

## 4 Discussion

We sought to examine the association between reproductive-related hormones with nicotine craving and use from late pregnancy through 12 weeks postpartum via secondary data analyses of an intensive longitudinal study among those with and without opioid use disorder. Overall, the results of this study offer three main takeaways. First, oxytocin was associated with nicotine craving and use during both prenatal and postpartum periods. Second, only during postpartum, estradiol was inversely associated with both nicotine craving and use. In contrast, progesterone did not have any meaningful associations with nicotine craving or use. Third, there was notable intra-individual variation in both craving and use, despite stable group-level means. Overall, these observations suggest that hormonal influences on the risk of perinatal nicotine use varies across individuals, perinatal timing, and hormone.

This is the first study to examine the association between oxytocin and nicotine use during the perinatal period. Oxytocin has recently been implicated as a possible protective factor against drug taking behaviors (Sanna and De Luca, 2021), with specific biological plausibility to protect against nicotine craving (Thanekar et al., 2026). However, the evidence to date is mixed. We observed that higher oxytocin values during pregnancy were associated with greater craving. This observation concurs with recent work demonstrating that exogenous administration of oxytocin was linked to heightened smoking urges in non-pregnant females but not males during acute abstinence, though it was not linked to smoking lapse in either sex (Simpson et al., 2024). In contrast, exogenous oxytocin has also been linked to reductions in cigarette consumption in laboratory-based settings in both non-pregnant females and males (Van Hedger et al., 2019). Further evidence suggests the influence of oxytocin may differ by context given oxytocin was not associated with stress-induced smoking (Van Hedger et al., 2020), yet significantly reduced cue-induced smoking (Miller et al., 2016). We also observed that a greater decline in oxytocin from late pregnancy to postpartum week four was linked to greater craving and use during the postpartum. Oxytocin plays a key role during the perinatal period contributing to labor, breastfeeding, and bonding/attachment (Walter et al., 2021b). In this context, a greater decline in oxytocin from pregnancy to postpartum may be more common in those who do not breastfeed given oxytocin increases with breastfeeding (Uvnäs­Moberg et al., 2020). However, it should be noted that oxytocin is released in pulses during the perinatal period (Uvnäs­Moberg et al., 2020), causing oxytocin values to potentially vary dramatically from moment to moment. Although we standardized the time-of-day collection, the single weekly measurements of oxytocin during the perinatal period may be insufficient for examining the association with nicotine-related outcomes. Oxytocin also contributes to the neurobiological reward system (Sanna and De Luca, 2021) suggesting there may be additional rationale for oxytocin to have a protective effect against drug-taking behaviors during the perinatal period. Overall, our observations suggest oxytocin may be linked to nicotine-related outcomes during the perinatal period but replication of our observations with more precise measurement is necessary.

Estradiol has been well studied in relation to nicotine use with ample strong evidence generated from preclinical models (Wetherill et al., 2016). While evidence from clinical research is more mixed, in general, estradiol is thought to increase the risk of nicotine use (Wetherill et al., 2016). Our observations contrast with this current theory, as we observed lower estradiol was associated with greater craving throughout late pregnancy and the early postpartum, and a lower risk of use during the early postpartum. We also observed a striking lack of meaningful associations between progesterone with nicotine use or craving, despite prior work observing a protective association of progesterone on postpartum nicotine-related outcomes. Specifically, two separate studies that conducted a preliminary evaluation of exogenous progesterone administration versus placebo for prevention of postpartum CC return-to-use prevention separately observed administration of exogenous progesterone during the postpartum period was associated with lower nicotine craving among those who quit smoking during pregnancy (Allen et al., 2016, Forray et al., 2017). In contrast and in line with our present observations, null observations have also been observed in evaluation of endogenous progesterone within non-pregnant females (Harrison et al., 2020, Novick et al., 2022b, Novick et al., 2022a, Pang et al., 2018, Schick et al., 2024, Tosun et al., 2019). One theory that may explain this seeming disconnect is that allopregnanolone, a metabolite of progesterone that has stress-reducing effects, is rapidly produced from exogenous progesterone and, therefore, may have greater bioavailability than occurs naturally (Diviccaro et al., 2022). Allopregnanolone has documented associations with nicotine-related outcomes in non-pregnant females (Allen et al., 2015, Novick et al., 2022b) and may also contribute to risk of postpartum depression (Reddy et al., 2023). Allopregnanolone has yet to be examined during the perinatal period in relation to nicotine use. Importantly, the present study is the first to examine the association between multiple hormones simultaneously and nicotine-related outcomes. A higher P:E2 ratio was linked to greater craving in the present study; in contrast prior research in non-pregnant females observed that an increasing P:E2 ratio was linked to a higher probability of smoking cessation (Saladin et al., 2015), though null observations have also been observed elsewhere in both pregnant and non-pregnant females (Allen et al., 2019, Harrison et al., 2025, Harrison et al., 2020). It is noteworthy that estradiol was the lone hormone that had a meaningful association with the nicotine-related outcomes both when examined independently, as well as in models accounting for other hormones. Overall, these observations suggest that estradiol may influence perinatal nicotine craving and use, but this influence may vary via the presence of other hormones, including progesterone. Additional research is needed to disentangle these complex hormonal relationships.

In general, reproductive-related hormones significantly increase during pregnancy followed by a dramatic decline quickly after childbirth. Given this pattern, the perinatal period offers a natural experiment to examine how changes in hormones may influence changes in nicotine use. While there are certainly enhanced motivations by pregnant and parenting individuals to abstain from nicotine use during and after pregnancy to reduce harms to their offspring (DeVito et al., 2021), hormones may contribute to this success (or lack thereof). We took inspiration from the growing body of evidence linking perinatal hormones with risk for postpartum depression (Osborne et al., 2017) to examine hormonal links during pregnancy only, postpartum only, and the change from pregnancy to postpartum. The postpartum analyses used paired weekly hormone levels with daily reports of craving in mixed-effects models, which used the full set of repeated observations and accounted for the correlation among observations contributed by the same participant. Relatedly, it is important to note that a large portion of our sample had opioid use disorder, and this may have also contributed to our observations. Chronic opioid exposure has been linked to adrenal insufficiency which alters the production of hormones (Li et al., 2020). Similarly, treatment and recovery from opioid use disorder may influence craving, metabolism, and use of nicotine (Oncken et al., 2020, Vlad et al., 2020). Thus, opioid use disorder may alter the observed relationships between hormones and nicotine craving and use. Given our small sample size, we did not have the statistical power to adjust for this variable in our between-subject analyses. This should be considered when interpreting these results. Overall, our observations suggest that the link between hormones and risk of nicotine use may have high variability both within and between individuals. Therefore, accurately interpreting between-subject associations may be more difficult, especially when important confounders are present. As technology advances, more opportunities to conduct detailed within-subject analyses on a micro level (e.g., across minutes or hours) will become available. This will enhance our understanding of the perinatal-specific opportunities to reduce nicotine consumption and can inform personalized medicine and just-in-time adaptive intervention approaches.

This secondary data analysis provides a novel exploration into the links between reproductive-related hormones and nicotine-related outcomes using both subject-level and repeated-measures analyses within an intensive longitudinal design, yet limitations are present. First, our sample was small and not generalizable to the general population. Of note, 78% of those in our study sample had opioid use disorder given the purpose of the parent study. Further, due to sample size limitations and multicollinearity among predictors, we were unable to adjust for multiple potential confounding variables in the analyses, such as breastfeeding status or stress levels/exposure, despite the known relationships between these variables (Ananth and Schisterman, 2017, Linde-Krieger et al., 2026, Yen and Jaffe’s, 2023). Based on our preliminary fundings, future fully powered studies should aim to disentangle complex relationships among these hormones, other contextual influences, and postpartum nicotine use/craving. Second, we did not include biochemical verification of nicotine use and relied on self-reported use. This may skew our observations towards the null due to non-differential misclassification bias via stigma and social desirability bias. Next, we pooled CC and ENDS use in our analyses for statistical power, though it is possible that hormonal influence may differ for these two forms of nicotine administration. Future research should examine these associations by type of nicotine use, as well as with dual CC and ENDS use. Lastly, we did not control for multiple comparisons in these exploratory analyses, which increases the likelihood of Type 1 error. Despite these limitations, the observations made in this study are novel and warrant further investigation in more diverse samples with more robust and rigorous measurement of both hormones and nicotine-related outcomes.

In conclusion, we observed several links between reproductive-related hormones and risk of perinatal nicotine use. Of highlight, oxytocin was positively associated with craving and risk of use throughout the perinatal period, whereas estradiol was associated with the same outcomes during the postpartum period only and directionality of this relationship varied when accounting for other hormones. It is possible that the presence of opioid use disorder, of which pertains to the majority of study participants had, may influence these observations, potentially explaining why our observations contrast with prior evidence. Given nicotine use is exceptionally high in those with opioid use disorder (Jarlenski et al., 2020), our observations may prove especially useful to future work that focuses on this high-risk population. Additional research is needed to examine within-subject variations in hormones and nicotine use across the perinatal period, and how these associations may be used to protect individuals from continued or return to use after pregnancy.

## CRediT authorship contribution statement

**James Baurley:** Writing – review & editing, Visualization, Software, Resources, Funding acquisition, Formal analysis, Conceptualization. **Allen Alicia M:** Writing – review & editing, Writing – original draft, Supervision, Resources, Project administration, Methodology, Investigation, Funding acquisition, Data curation, Conceptualization. **Xinger Xia:** Writing – review & editing, Methodology, Investigation. **Whitney Norris:** Writing – review & editing, Methodology, Investigation. **Arushi Chalke:** Writing – review & editing, Visualization, Methodology, Data curation. **Linde-Krieger Linnea:** Writing – review & editing, Methodology, Investigation, Funding acquisition, Data curation, Conceptualization. **Clawson Ashley:** Writing – review & editing, Methodology, Investigation.

## Ethics declaration

Written informed consent to take part in the study and to publish the article has been obtained from all participants or their legal representatives. The privacy rights of participants have been observed.

This study was performed in compliance with relevant laws, regulatory frameworks and guidelines where the research took place. This study was approved by the University of Arizona. (Approval No. 2009060851R001)

## Sources of funding

Arkansas Children’s Research Institute and the National Center for Opioid & Addiction Research (NCOAR) at Arkansas Children's Research Institute (ACRI), Eunice Kennedy Shriver National Institute of Child Health & Human Development (DP2HD105541), 10.13039/100006108National Center for Advancing Translational Sciences (UL1TR000445), National Institute of Minority Health and Health Disparities Institute (P50MD017319).

## Declaration of Competing Interest

One author (Allen) is an editorial board member for this journal and was not involved in the editorial review or the decision to publish this article. There are no other declarations of interest for any author.

## Data Availability

Upon reasonable request made to corresponding author.
